# Genome Sequence of *Pasteurella multocida* Razi 0002 of Avian Origin

**DOI:** 10.1128/genomeA.00161-17

**Published:** 2017-04-13

**Authors:** Lisa D. Sprague, Keyvan Tadayon

**Affiliations:** a Friedrich-Loeffler-Institut, Federal Research Institute for Animal Health, Institute for Bacterial Infections and Zoonoses, Jena, Germany; b Department of Microbiology, Razi Institute, Agriculture Research, Education and Extension Organization, Karaj, Iran

## Abstract

We report here on the genome sequence of *Pasteurella multocida* Razi 0002 of avian origin, isolated in Iran. The genome has a size of 2,289,036 bp, a G+C content of 40.3%, and is predicted to contain 2,079 coding sequences.

## GENOME ANNOUNCEMENT

This paper announces the *de novo* genome sequence of *Pasteurella multocida* vaccine strain Razi 0002, isolated in Iran (1, 2). Genomic DNA was isolated from an ethanol-inactivated overnight culture grown in tryptose phosphate broth with a Qiagen Genomic-tip 100/Q and the genomic DNA buffer set (Qiagen, Hilden, Germany). DNA quality was examined by using both a NanoDrop spectrophotometer (Thermo Scientific, Schwerte, Germany) and a Qubit 2.0 fluorometer (Life Technologies, Inc., Darmstadt, Germany). Whole-genome sequencing was performed on the RS sequencer with SMRT technology PacBio RSII (Pacific Biosciences, Menlo Park, CA, USA) at GATC Biotech (Constance, Germany), using standard protocols according to the manufacturer’s instructions, which were followed throughout the sequencing process (3). *De novo* assembly was performed with SMRT Portal version 2.3.0 (Daemon version 2.3.0, SMRT View version 2.3, and SMRTpipe version 2.3.0; Pacific Biosciences) from 25 contigs, with an *N*_50_ contig length of 2,289,036 bp. The genome has a size of 2,289,036 bp and a G+C content of 40.3% (Geneious 9.0.5; Biomatters, Auckland, New Zealand). Genome annotation was done using the NCBI Prokaryotic Genome Annotation Pipeline (http://www.ncbi.nlm.nih.gov/genome/annotation_prok/) and predicted 2,158 genes, 2,079 coding sequences (CDSs), 176 pseudogenes, 56 tRNA genes, 19 rRNA genes, and four noncoding RNA (ncRNA) genes.

### Accession number(s).

The sequence of *Pasteurella multocida* Razi 0002 has been deposited at DDBJ/EMBL/Genbank under the accession no. CP019081.

## References

[1] Tavasoli A, Sotoodehnia A, Aarabi I, Vandyousefi J 1984 A case report of fowl cholera disease in north of Iran. Arch Razi Inst 34:39–41.

[2] Sotoodehnia A, Aarabi I, Vandyousefi J, Tavasoli A 1984 The efficacy of the autogenous fowl cholera killed aluminum hydroxide vaccine in ducks in Iran. Arch Razi Inst 34:71–74.

[3] Eid J, Fehr A, Gray J, Luong K, Lyle J, Otto G, Peluso P, Rank D, Baybayan P, Bettman B, Bibillo A, Bjornson K, Chaudhuri B, Christians F, Cicero R, Clark S, Dalal R, Dewinter A, Dixon J, Foquet M, Gaertner A, Hardenbol P, Heiner C, Hester K, Holden D, Kearns G, Kong X, Kuse R, Lacroix Y, Lin S, Lundquist P, Ma C, Marks P, Maxham M, Murphy D, Park I, Pham T, Phillips M, Roy J, Sebra R, Shen G, Sorenson J, Tomaney A, Travers K, Trulson M, Vieceli J, Wegener J, Wu D, Yang A, Zaccarin D, Zhao P, Zhong F, Korlach J, Turner S 2009 Real-time DNA sequencing from single polymerase molecules. Science 323:133–138. doi:10.1126/science.1162986. 19023044

